# The Durability of an Organic–Inorganic Sol–Gel Interlayer in Al-GFRP-CFRP Laminates in a Saline Environment

**DOI:** 10.3390/ma12152362

**Published:** 2019-07-25

**Authors:** Barbara Surowska, Monika Ostapiuk, Patryk Jakubczak, Magda Droździel

**Affiliations:** Department of Materials Engineering, Faculty of Mechanical Engineering, Lublin University of Technology, 36 Nadbystrzycka St., 20-618 Lublin, Poland

**Keywords:** sol–gel coating, FML, EIS, corrosion

## Abstract

The aim of the study was to assess the selected properties of a hybrid organic–inorganic silane sol–gel coating (HSG) used in hybrid fiber metal laminates (FML) in a corrosion environment. The HSG coating on the aluminum alloy was produced using 3M™ AC130-2 formulation consisting of 3-glycidoxypropyl-trimethoxysilane (GPTMS) and tetra-n-propoxyzirconium (zirconium(IV) propoxide) (TPOZ). Laminates consisted of aluminum alloy AA2024-T3 sheets, with carbon fiber reinforced polymers (CFRPs) and a glass fiber reinforced metal–composite structure (GFRP). Potentiodynamic and polarization curve and impedance (EIS) tests were carried out on HSG at ambient temperatures after 1 h and 150 h of soaking. Neutral 0.5 M NaCl and 0.8 M NaCl solutions were used for open circuit potential (OCP) and potentiodynamic tests, and 0.5 NaCl was used for the EIS test. A neutral salt spray (NSS) test was applied to laminates with a 12 week exposure period. The results obtained revealed that the HSG coating did not provide sufficient protection against corrosion of the aluminum alloy in direct contact with an aggressive environment but was effective as an interlayer. Local aluminum sheet perforation did not lead to delamination at the metal–composite interface regardless of the type or configuration of the composite. This confirms the durability of HSG used in FMLs.

## 1. Introduction

The development of fiber reinforced polymer (FRP) composites enables the design of lighter engineering metal–composite structures. The joining of FRP elements by adhesive bonding and the introduction of fiber metal laminates (FML) has intensified studies on the physicochemical properties of surface and metal–composite interfaces in the context of adhesion and metal corrosion. One relatively safe combination is a glass fiber reinforced metal–composite structure (GFRP), as used in GLARE (Glass Laminate Aluminum Reinforced Epoxy) laminates [1]. In such materials, corrosion, leading to delamination, occurs at the interface of the aluminum alloy and the composite as a result of humidity penetrating the unprotected edges. Moreover, a pitting corrosion of metal can occur due to insufficient protection of the external surfaces, with a risk of perforation when 0.3–0.5 mm sheets are used [2,3]. Carbon fiber reinforced polymers (CFRPs) are more effective construction materials in terms of their mechanical and physical properties. However, they belong to conducting materials, which create a galvanic cell in contact with aluminum, with the metal acting as the anode. Research on the corrosion of such combinations is conducted in terms of their application in the aircraft industry [4,5], electrical engineering [6,7], sea water engineering [8], and other fields. One important factor, apart from reducing galvanic corrosion in aluminum-CFRP laminates, is the adhesion of the two components [9,10,11]. This is why the aluminum surface preparation method and the barrier properties of the interlayer must be selected on the basis of these two aspects.

The AA2024 aluminum alloy, widely used in the aircraft industry, requires protection against corrosion. The surface of elements that come into direct contact with the environment usually have a conversion coating applied or undergo anodizing and receive an intermediate layer (primer) and paint coating [9,10,12]. Chromate conversion coatings (CCC) and oxide coatings produced during chromic acid anodizing (CAA) are considered to provide good protection against corrosion but are currently being withdrawn due to the toxicity of chromium(VI) [13,14]. For this reason, alternative corrosion protection methods are being introduced for metal surfaces. At the same time, these methods result in high adhesion to FRPs. One such method involves the use of functional silane coatings produced via the sol–gel process. Silane adhesion promoters contain alkoxy groups capable of hydrolysis and bonding with any non-organic material containing hydroxyl groups on its surface and functional groups, which react with the polymer’s functional groups. They may also act as curing agents in processes involving humidity and surface modifiers [15,16]. Hybrid organic–inorganic sol–gel coatings with corrosion protection properties that polymerize at low temperatures are a closely studied group and have been implemented in engineering for over a decade [15,16,17,18,19,20,21,22,23]. The most frequently tested materials for the protection of aluminum alloys are based on 3-glycidoxypropyl-trimethoxysilane (GPTMS) combined with tetra-n-propoxyzirconium (zirconium(IV) propoxide) (TPOZ) (about 72% and 12%, respectively, according to [15]). These second-generation coatings ensure a high corrosion resistance by creating a dense –Si–O–Si– network and providing high adhesion to metal surfaces, owing to the formation of covalent Me–O–Si bonds [17,18].

Metal-based precursors demonstrate high activity for the epoxide ring opening of organically functionalized silanes like GPTMS. The addition of Zr promotes the formation of a more amorphous Si–O–Si network instead of the ring (Si–O)_4_ unit [19,20]. TPOZ causes an increase in primary bond density across the metal–hybrid interface due to the formation of Zr–O–X bonds, and increases organic crosslinking resulting from the ability of TPOZ to catalyze the opening of the epoxy rings of GPTMS [21]. The opened epoxy ring can form a C–O–C bond. An epoxy is a functional group that can form a polymerized species. [19]. Purcar et al. [20] used GPTMS with TPOZ as an inorganic precursor to obtain the modified coatings with nanoparticles of ZnO and proposed a probable reaction mechanism that can occur when ZnO is modified using a silane precursor.

Fontinha et al. [16] showed that an intermediate oxide layer is formed on the surfaces of aluminum alloys, formed by Al–O–Si and Al–O–Zr bonds as a result of condensation reactions between Zr–OH and Si–OH groups, with Al–OH groups of the native oxide layer present in the metal surface. Rodic et al. [22] showed that the bonding between silicon, oxygen, and zirconium (Si–O–Si, Si–O–Zr) may be crucial to achieving excellent corrosion protection for aluminum alloys. Still, these protective properties have been observed to decrease under long-term environmental impacts. Purcar et al. [23] concluded that Al–OH groups can play the role of a catalyst in organic chain degradation. Therefore, corrosion inhibiting additives [24] or primers with corrosion inhibitors are proposed for the direct protection of aluminum surfaces against the natural environment [25,26].

This paper presents the results of a study on a hybrid organic–inorganic silane sol–gel coating (HSG) used as an interlayer in aluminum alloy-carbon and glass fiber reinforced epoxy composite connections in terms of resistance to the direct and indirect impact of humid environments at different levels of aggressiveness.

## 2. Materials and Methods

### 2.1. Materials

The organic–inorganic hybrid sol–gel coatings on the aluminum alloy AA2024-T3 (AlCu4Mg1) were produced using the 3M™Surface Pre-Treatment AC 130-2 (3M, Minneapolis, MN, USA) two-component formulation (sol-gel coatings were produced at the Lublin University of Technology, Lublin, Poland), which is designed to improve the adhesive bonding of metal and composite surfaces. This water-based and non-chromated sol–gel formulation consisted of a 3-glycidoxypropyl-trimethoxysilane (GPTMS) and zirconium(IV) propoxide solution (TPOZ) [25,27]. According to 3M Safety Data Sheet], Part A consists of <1% wt TPOZ as aqueous solution, and Part B consists of >97% wt GPTMS in methyl alcohol. In order to prepare the organic–inorganic sol, the volume fraction of Part A and Part B was about 49:1 The molecular and structural formulas of both precursors are presented in Table 1. Small amounts of glacial acetic acid (GAA) lowered the pH level of the solution to 3.8 [25,27].

**Figure 1:**
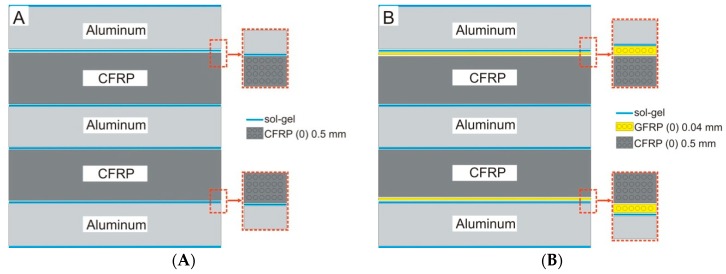

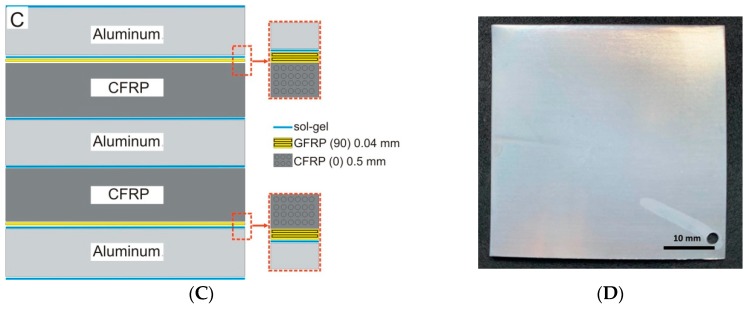
Sample configuration schemes: (**A**) aluminum and carbon fiber reinforced polymer (Al-CFRP), (**B**) aluminum and glass fiber reinforced metal–composite structure (Al-GFRP)(0)-CFRP, (**C**) Al-GFRP(90)-CFRP, and (**D**) the true sample, top view.

The surface of the metal sheet with a thickness of 3 mm was delivered with a protective surface film. After film removal, the surfaces were pre-degreased with acetone. Then, manual scrubbing was carried out using a general-purpose abrasive pad Scotch-Bride 07447+ (3M). The scrubbing was carried out in two perpendicular directions until a homogeneous grinded surface was obtained. The next step was to thoroughly degrease the surface with acetone and carry out a water-break-free surface test. A thin layer of sol was applied using a brush. Next, the samples were air dried for 60 min. In the case of a higher number of layers, subsequent ones were applied after drying. Single, double, and triple layer sol–gel coatings were applied for electrochemical testing and double layer for the FML production. The uncoated aluminum alloy was used as reference material in electrochemical examinations. FMLs were produced for salt spray testing. Three types of FMLs were studied: Al-CFRP, Al-GFRP-CFRP with the same fiber direction, and one with opposite fiber directions, respectively (Figure 1). The composite materials used for metal bonding were unidirectional epoxy matrix prepregs reinforced with high strength carbon fiber (0.135 mm thick, produced by NTPT, Renens, Switzerland) and S-type glass fiber (ultra-thin, 0.04 mm thick, produced by NTPT) [28]. The panels were manufactured by the autoclave method with vacuum bag assistance (SCHOLZ Maschinenbau autoclave, Coesfeld, Germany). The curing was a two-step temperature process (80 °C for 1 h and 150 °C for 4 h). The heating and cooling rate was 1.2 °C/min. The pressure was 0.6 MPa and the vacuum was 0.02 MPa throughout the entire process [28]. The external surface of the FML samples (50 × 50 mm) was covered with a double sol–gel coating without a top-coat. The edges were secured with foil.

### 2.2. Methods

The open circuit potential (OCP), polarization curve and impedance (EIS) of the coatings were measured using an Atlas 0531 potentiostat (Atlas-Sollich, Rebiechowo, Poland) with an FRA (Frequency Response Analyzer) and subjected to direct analysis in the measurement program. The three-electrode cell consisted of a saturated calomel reference electrode (SCE), a platinum foil electrode, and the test sample as the working electrode in the horizontal position. The exposed area was 2.27 cm^2^. Neutral 0.5 M NaCl and 0.8 M NaCl solutions were used for the OCP and potentiodynamic tests. Three repetitions were performed, and the standard deviation was calculated. The EIS tests were carried out in a neutral 0.5 M NaCl solution and at ambient temperature after 1 h and 150 h of soaking. The amplitude of the sinusoidal signal was 10 mV versus the open circuit potential, and the frequency span was 100 kHz–0.01 Hz. The impedance spectra are presented in the form of Bode and Nyquist plots.

The test in a salt chamber (ASCOT CC450 xp, Ascott Analytical Equipment Limited, Staffordshire, UK) was carried out on FMLs in 0.8 M NaCl at a temperature of 37 °C and 95% relative humidity (RH) for 12 weeks according to EN ISO 9227 [29], with a modification after a preliminary test. Two samples were taken out after each week. The residues of the spray solution and the corrosion products were removed by light mechanical cleaning treatment under running water according to EN ISO 9227 [29] and EN ISO 8407 standards [30]. The chemical cleaning was performed by immersion in 10% HNO_3_ aqueous solution for about 40 seconds in RT for specimens with visible deposits of aluminum hydroxide. The weight was measured with a laboratory balance (Radwag WAS 220/X, Radwag Wagi Elektroniczne, Radom, Poland) to an accuracy of 0.0001 g. The mass loss was calculated as the difference between the final and initial mass, with the exposed surface area of each sample taken into consideration. 

The macro- and microstructures prior to and after the corrosion tests were examined with a light microscope and a scanning electron microscope (NovaNanoSEM 450, FEI Company Japan Ltd., Tokyo, Japan).

## 3. Results and Discussion

### 3.1. Microstructure and Electrochemical Properties of Sol–Gel Coatings

**Figure 2:**
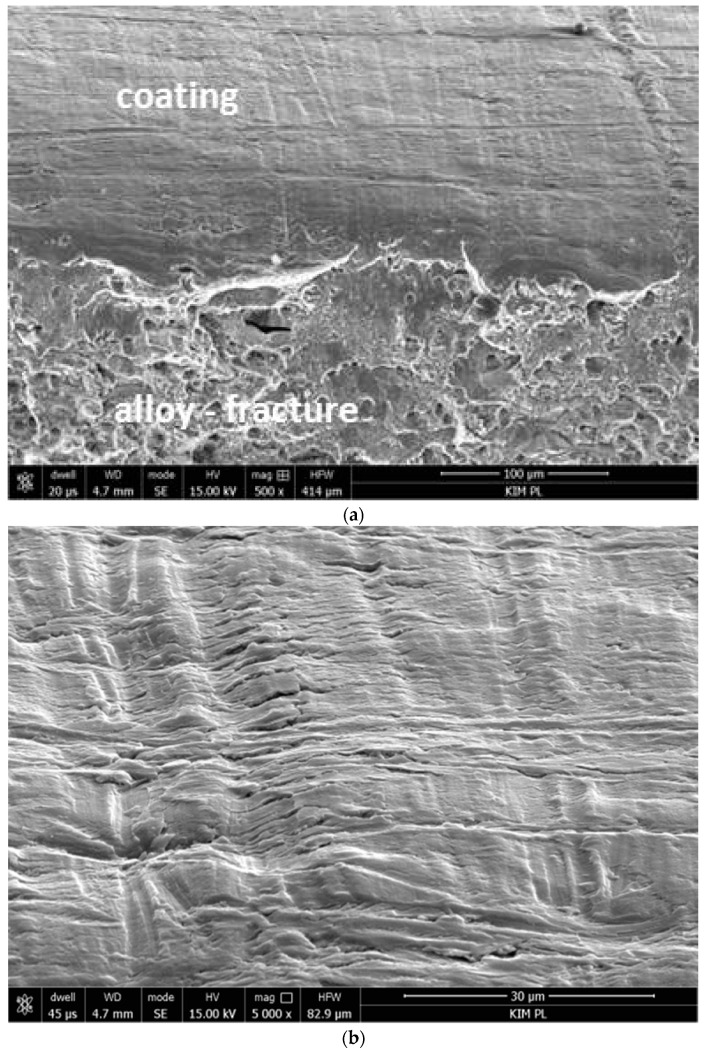
Microstructure of the sol–gel coating on the aluminum alloy: (**a**) perspective view on the fracture, (**b**) top view; SE images SEM.

**Figure 3:**
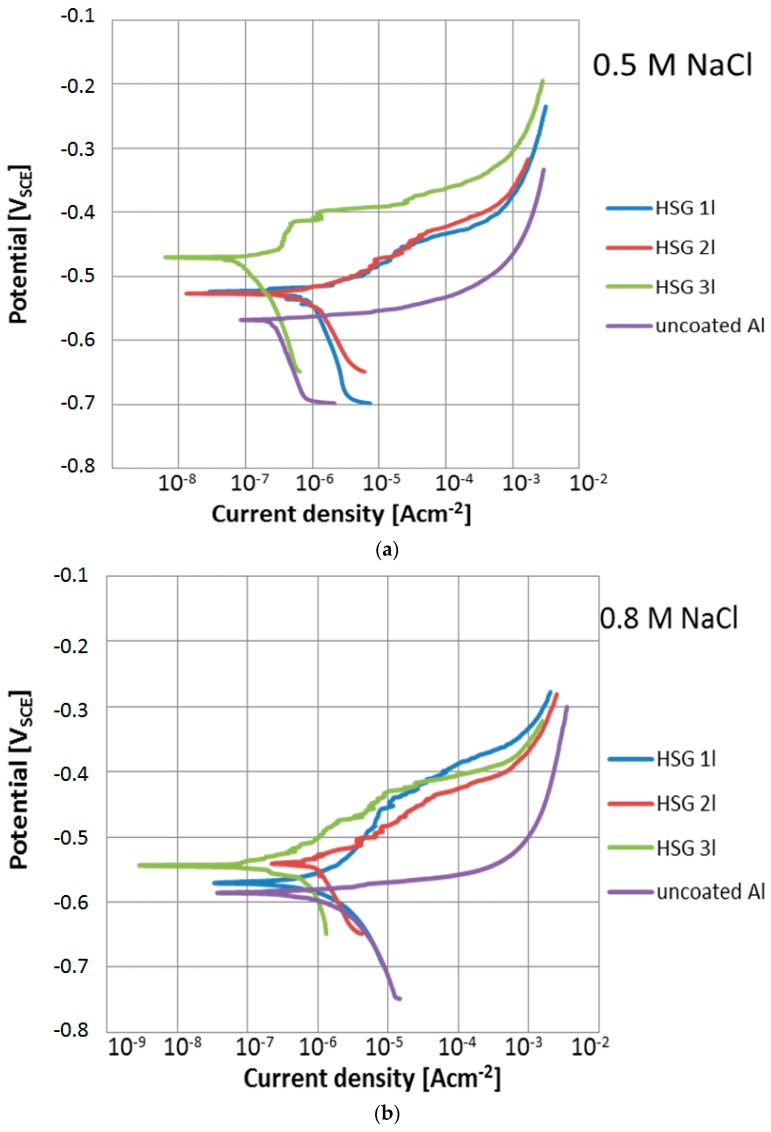
Potentiodynamic curves of uncoated and coated aluminum alloy in (**a**) 0.5 M NaCl and (**b**) 0.8 M NaCl.

The HSG coating on the AA2024 was uniform, compact, and reflected the surface topography (Figure 2). The thickness and electrochemical parameters of the coating are summarized in Table 2, and the polarization curves are presented in Figure 3.

The single-layer coating featured the lowest corrosion resistance in the potentiodynamic tests. The triple-layer coating exhibited the highest properties (Figure 3). The very low levels of corrosion current *i*_corr_ and high polarization resistance *R*_p_ (Table 2) of this coating resulted from its thickness and morphology.

**Figure 4:**
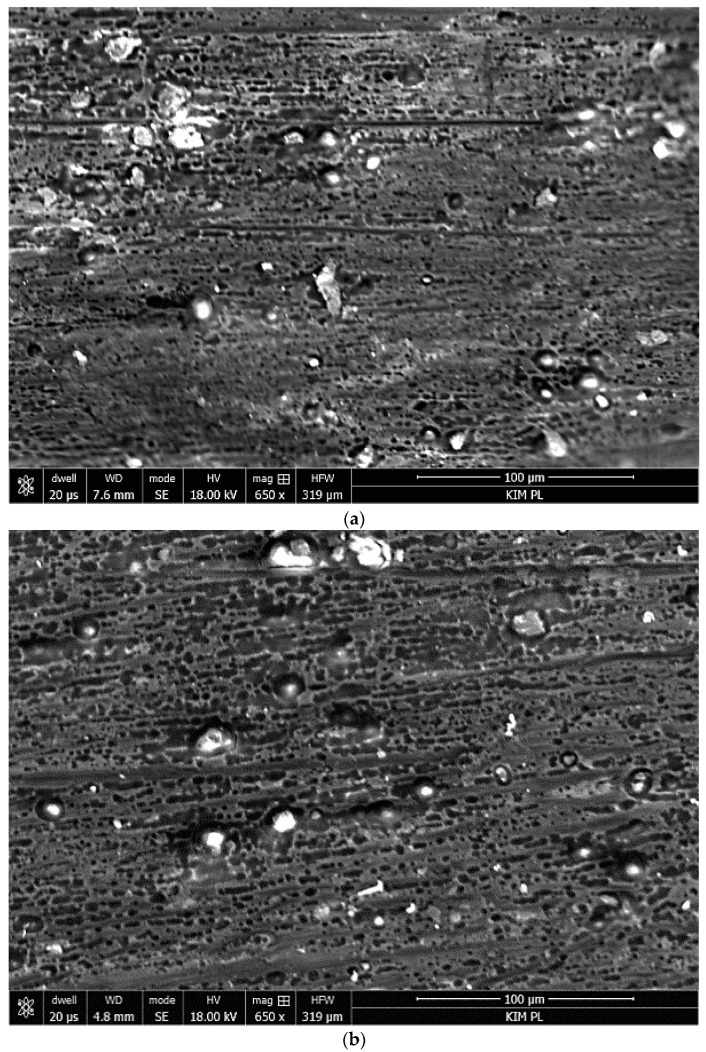

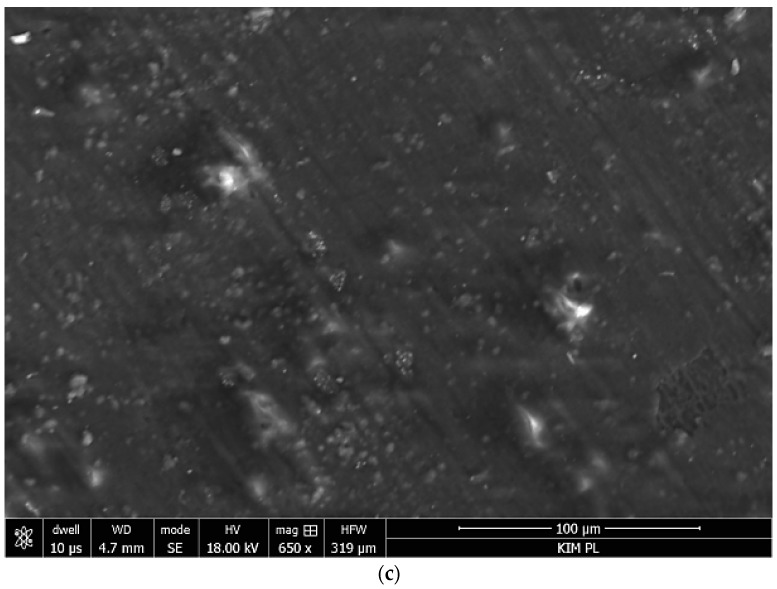
Microstructure of the coatings on aluminum surface after the potentiodynamic test: (**a**) single-layer coating, (**b**) double-layer coating, and (**c**) triple-layer coating; 0.5 M NaCl solution; SE images SEM.

This coating was still smooth after the test (Figure 4c), while thinner coatings became porous with uncovered intermetallic phase precipitates visible (Figure 4a,b). This proved that water was absorbed and chlorine ions penetrated the coating. Although increasing the solution concentration aggravated the degradation of the coating, the least significant changes in the properties were observed for the double-layer coating. The thickest coating retained the best electrochemical parameters (Figure 3, Table 2).

**Figure 5:**
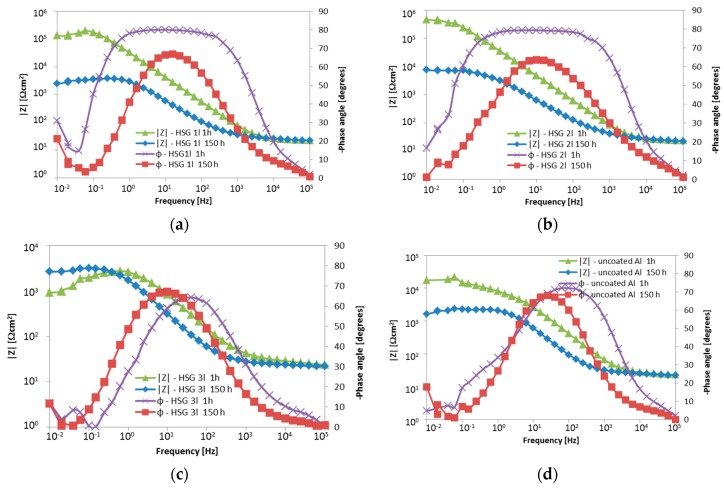
Bode plots of the hybrid organic–inorganic silane sol–gel coating (HSG) coated and uncoated aluminum alloy after 1 h and 150 h of soaking: (**a**) single-layer coating, (**b**) double-layer coating, (**c**) triple-layer coating and (**d**) uncoated AA2024; 0.5 M NaCl.

**Figure 6:**
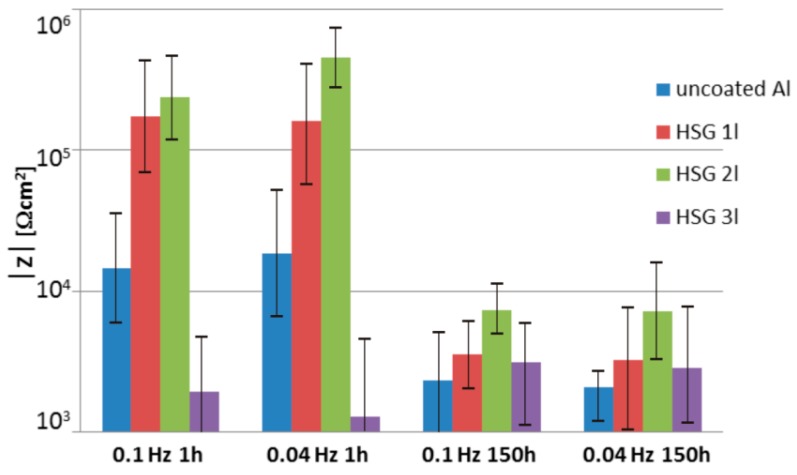
Comparison of the impedance modulus of HSG coatings and an uncoated aluminum alloy at 0.04 Hz and 0.1 Hz.

**Figure 7:**
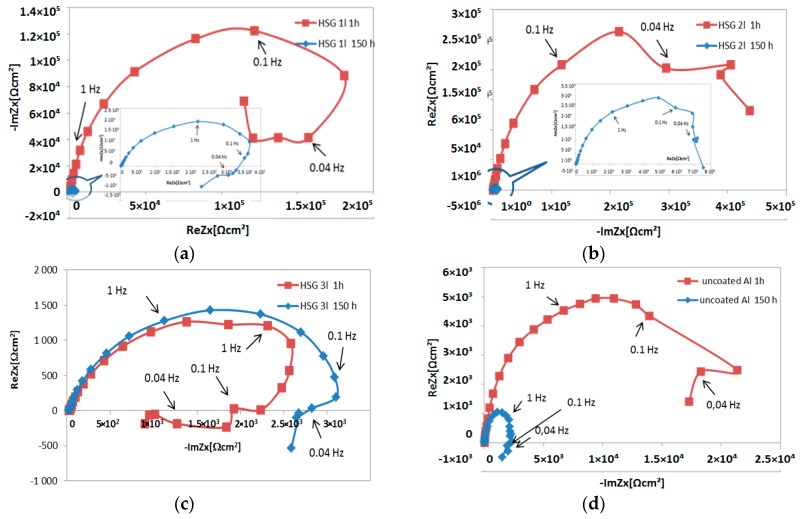
Nyquist plots of HSG coatings and uncoated aluminum after 1h and 150 h of soaking: (**a**) single-layer coating, (**b**) double-layer coating, (**c**) triple-layer coating, and (**d**) uncoated aluminum alloy.

The impedance measurements results are presented as Bode plots (Figure 5), featuring a comparison of the impedance modulus for low frequencies (Figure 6), and as Nyquist plots (Figure 7).

The impedance modulus versus frequency plots indicated the occurrence of a single time constant at the middle frequencies (Figure 5), which decreased with increasing immersion time. This demonstrated that the coatings underwent degradation over time. The triple-layer coating (Figure 5c) and uncoated alloy plots (Figure 5d) were indicative of a second time constant at low frequencies of around 0.1 Hz, corresponding to corrosion. At the initial state (1 h after immersion), the impedance modulus for low frequencies (below 0.1 Hz) of the single- and double-layer coating was of the order 10^5^ Ω cm^2^, whilst the value for the triple-layer coating was about 10^3^–5 × 10^4^ Ω cm^2^ and was comparable to the value obtained for the AA2024 alloy. After 150 h of immersion, the impedance modulus decreased by over one order of magnitude within the low frequencies for all samples except for the triple-layer coating, exceeding the values obtained for the uncoated alloy (Figure 6).

The phase angle plots (Figure 5) confirmed a single time constant within the middle frequencies for the uncoated alloy and triple-layer coating at both 1 h and 150 h immersion. At the same time, in the case of the single- and double-layer coating, the broad phase angle peaks (0.25–400 Hz and 0.63–400 Hz, respectively) after 1 h indicated two similar time constants, i.e., two processes taking place during immersion. The middle frequency range time constant is most likely related to the reactive epoxy-silane layer, with the lower frequencies related to the conversion Al-O-Zr interlayer. After 150 h, the shape and position of the maximum peak were comparable to those with the triple-layer coating. The visible reduction of the phase angle within the middle frequency range may be attributed to the porous structure of the cured sol–gel, as water may enter it and cause hydrolysis to occur. The disappearance of the second time constant may stem from interlayer degradation.

The Nyquist plots (Figure 7) indicate the complexity of the processes taking place both during the first stage after immersion in the solution and after 150 h. The semi-circle for the high and middle frequencies proves that the kinetics of the corrosion mechanism of each configuration is controlled regardless of the coating thickness by the rate at which the charge is transferred over the phase interface, while the contribution of the remaining processes (migration, convection, and diffusion) are negligible. A loop observed for low frequencies is usually interpreted as an LF (low frequency) inductive loop. Negative resistance may result from feedback between digestion and passivation, which take place at the same time. The occurrence of negative resistance in the impedance spectrum could also be connected to the processes developing near the alloy surface and not necessarily on the surface. The products of the degradation of the coating with poor adhesion to the alloy surface form a thick depletion layer and cause the charge transfer resistance to increase.

Such a shape on the Nyquist plot appears, for example, in the case of anodized aluminum alloys and some sol–gel coatings in NaCl solutions with inhibitors. The majority of the authors consider that the LF loop is related to the ionic relaxation phenomenon or absorption behavior [26,27,28]. According to Nowosu et al. [31] the presence of the LF inductive loop may be attributed to the relaxation process obtained by adsorption of the electrons of the conjugated O^2−^ systems on the electrode surface or the adsorption of the inhibitor on the electrode surface, which creates a barrier to current flow or to charge-transfer. Murthy [32] supposes this loop may be attributed to the relaxation process in the OH^−^_ads_ adsorbed species present on the surface of the samples. According to Charith et al. [33], in research conducted in a HCl solution with a corrosion inhibitor, the LF inductive loop is formed because of the relaxation of the bulk or surface species in the protective oxide film, and it may also be due to the adsorbed inhibitor molecule relaxation over the aluminum surface or due to Cl^–^ ion incorporation, charged intermediates, and O^2–^ ions on and inside the protective layer of the oxide. In their work on the impact of the morphology of the anodized layer on corrosion in 0.5 M NaCl after galvanostatic polarization, Liu et al. [34] proposed that the inductive loop reveals that the surface area is partially or totally active. Similarly, Veys-Renaux et al. [35] obtained loops in low frequencies in their in situ EIS measurements of an anodized aluminum alloy. Liu et al. [36] found that a low frequency inductive reactance loop may suggest a corrosion process under activation control. Klotz [37] thinks that one possible explanation for a low frequency loop is a two-step reaction into the intermediate state. That state could represent reaction sites, energy states in the band diagram, the occupancy of a reactive surface, or the concentration of the reaction product in a given volume. Charge carriers are removed from the intermediate state during second step of the reaction. The secondary effect must be much slower than the main reaction in order to produce a low frequency loop. In the Sol–Gel Handbook [38], the impedance spectra for sol–gel coatings are described as follows: a spectrum consisting of the HF domain (protective barrier), MF range (oxide layer), and LF range (onset of corrosion). For the AA2024-T3 alloy, the LF time constant is usually attributed to pitting corrosion. Yasakau et al. [39] concluded that the capacitive response of the sol–gel coating appears at high frequencies of about 10^5^ Hz. The relaxation process at middle frequencies (10^−1^–10 Hz) is ascribed to the intermediate oxide layer formed by alumina, zirconia, and silica. The time constant at low frequencies of <10^−1^ Hz is associated with the corrosion activity.

Summing up, the impedance spectra obtained during the EIS tests indicate progressive coating degradation in contact with an aqueous solution containing chloride ions. The first stage features hydrolysis of the polysiloxane network to monosilic acid Si(OH)_4_ [40]. Cl^−^ ions penetrate the porous coating and cause pitting corrosion in the aluminum alloy when they reach the metal-coating interface [41]. During pitting corrosion, the anodic reaction produces aluminum hydroxide in the following reaction:

Al^+3^ + H_2_O → [Al(OH)]^+2^ + H^+^ + e^−^. (1)

This follows the formation of [Al(OH)]^+2^ complex which will react with the chloride ion to form a soluble complex as follows [42]:

[Al(OH)]^+2^ + Cl^−^ → [Al(OH)Cl]^+^ (2)

[Al(OH)Cl]^+^ + H_2_O → Al(OH)_2_Cl + H^+^ (3)

**Figure 8:**
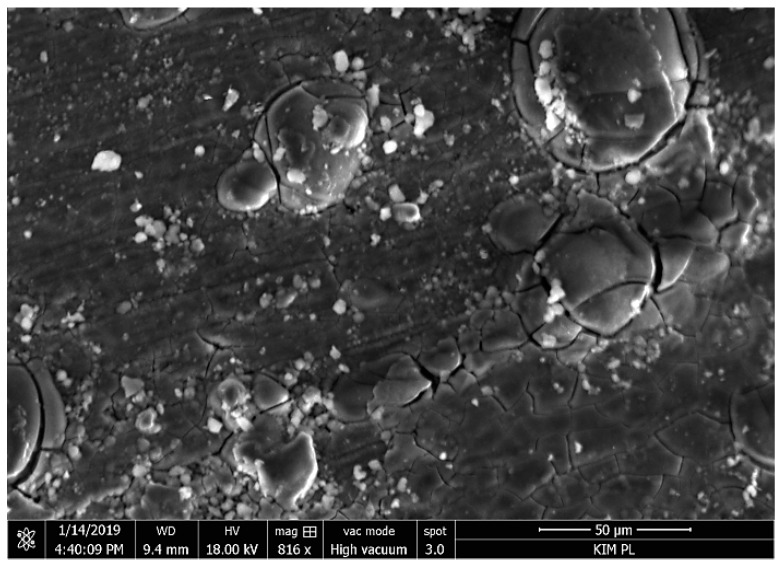
Microstructure of the sol–gel coating after 150 h of soaking in 0.5 M NaCl; SEM.

Blisters and loss of continuity in the coating were observed as a result of corrosion products forming on samples with the thickest coating after 150 h of soaking (Figure 8). A similar morphology of the degraded coating was observed by Gobara et al. [42] and Poznyak et al. [43].

The coating characteristics obtained in direct contact with an aggressive environment demonstrate the material’s poor durability in conditions where there is a loss of barrier capability of the top coat. As the intention was to use HSG instead of anodizing aluminum in FML production, considering the high adhesive properties and alleged effective protection against corrosion offered by the coating, it was necessary to test the durability of the metal–composite interface following a loss of the corrosion protection in laminates.

### 3.2. Salt Spray Test of Laminates

**Figure 9:**
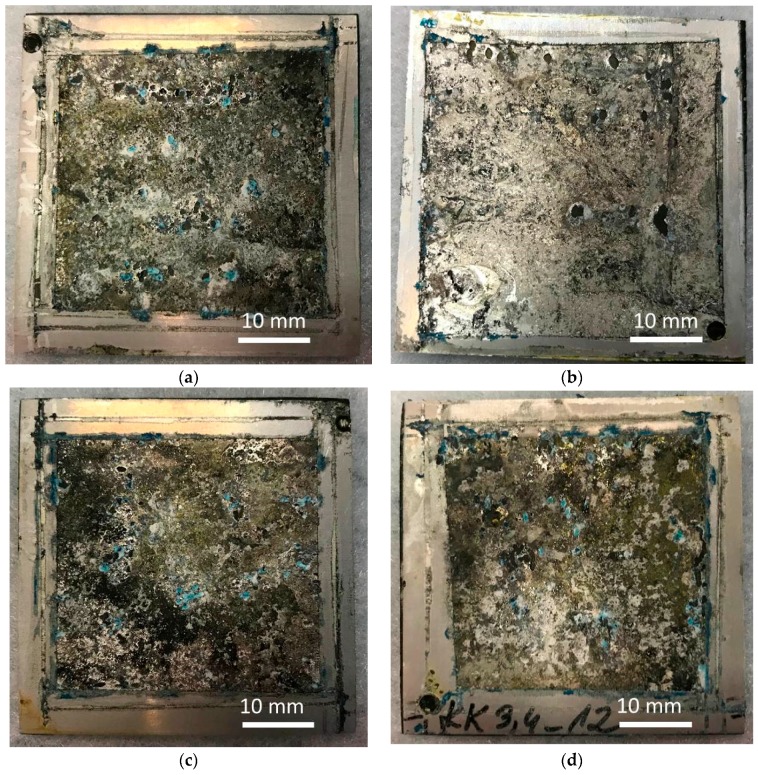

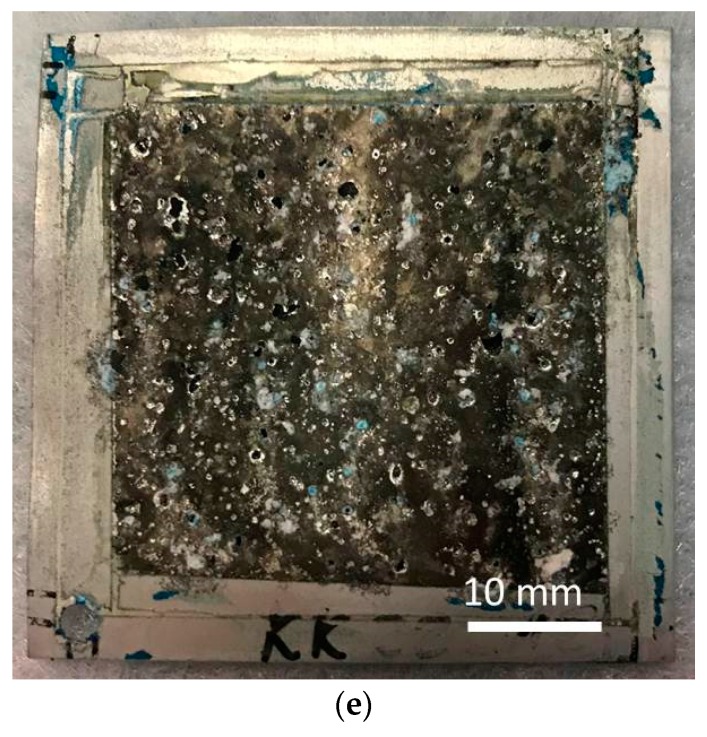
Laminate surface macrostructure: (**a**) Al-CFRP, (**b**) Al-CFRP reverse side, (**c**) Al-GFRP(0)-CFRP, (**d**) Al-GFRP(90)-CFRP, and (**e**) an aluminum sheet, after 12 weeks of testing in a salt chamber; 0.8 M NaCl.

Due to the activity of the salt spray, the external coating on all samples underwent degradation, and metal corrosion process ensued (Figure 9). The surface was covered with the products of aluminum corrosion and coating degradation. Pitting could be seen across the entire surface of the samples on the sprayed side. On the reverse side, the surface suffered minor degradation, with pits being less numerous but having large diameters (Figure 9b). Degradation of this coating type was also observed by Gobara et al. [42], Poznyak et al. [43], and Capelossi et al. [44] who, after a six-week salt spray test of anodized AA2024 alloy with HSG, determined the presence of sol–gel only in the pores of the anodized layer.

**Figure 10:**
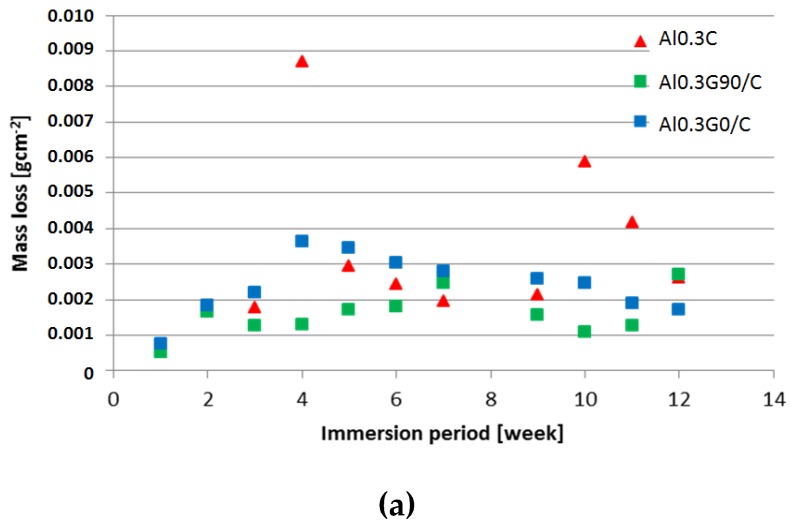

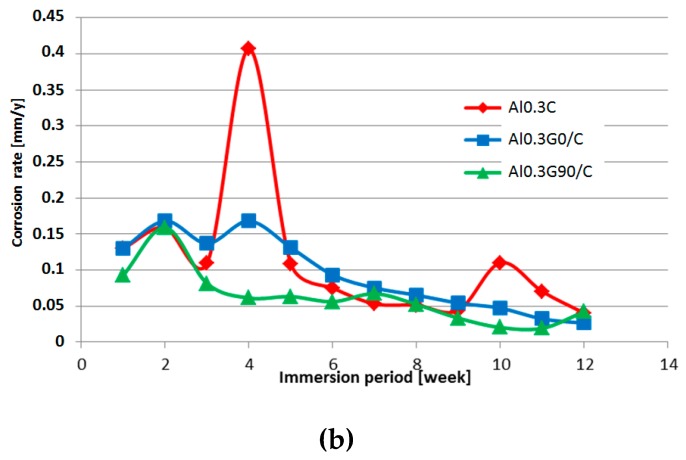
Mass loss of the laminates during the 12 week test (**a**) and corrosion rate (**b**); 0.8 M NaCl;.

The impact of the laminate layer configuration on the pitting corrosion level was not visible by macroscopic observation. The measurements of mass loss over time indicated a relatively constant corrosion ratio in the hybrid laminates with GFRP layers (Figure 10). A local increase in mass loss was observed in laminates without GFRP, which may be connected to galvanic corrosion after the perforation of the aluminum layers. The calculated corrosion rate decreases with an increase of immersion time independent from laminate configuration, probably because the corrosion products act as protection barrier. Because of the small number of samples and the significant spread of the results, a quantitative comparative analysis of the corrosion ratio for different laminate configurations was not possible.

**Figure 11:**
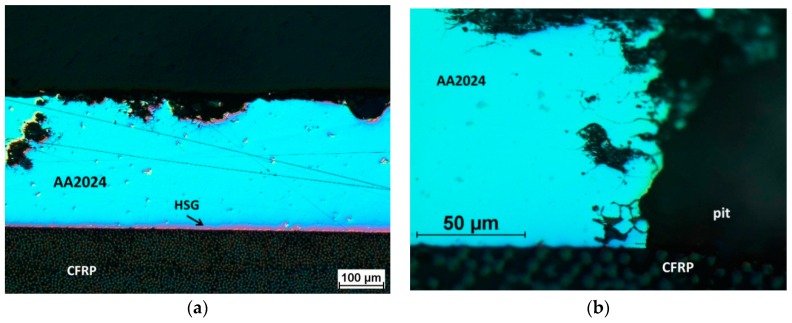
Aluminum alloy corrosion (**a**) and pitting area (**b**) in the Al-CFRP laminate after 12 weeks in a salt chamber; LM, Nomarski interference contrast.

**Figure 12:**
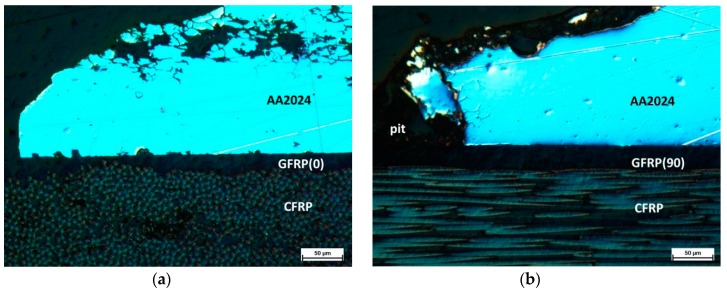
Laminate microstructure: **a**) Al-GFRP(0)-CFRP and **b**) Al-GFRP(90)-CFRP after 12 weeks in a salt chamber; LM, Nomarski interference contrast.

Microscopic observation of the interface on sections of the pitting areas was performed to identify the corrosion mechanism at the metal–(HSG)–composite interface. Examples of representative structures are shown in Figure 11 and Figure 12.

In the Al-CFRP laminate, in areas located at some distance from the pitting, the metal–composite interface was continuous, without delamination, which showed the good adhesion obtained with HSG and a lack of galvanic corrosion (Figure 11a). Intercrystalline corrosion was visible in the pitting area of the aluminum alloy, the metal–composite interface showed no signs of interlayer degradation, and the connection lasted for 12 weeks (Figure 11b). In laminates with a glass composite layer, intercrystalline corrosion was observed to progress in the metal in addition to pitting corrosion (Figure 12). In the Al-GFRP(0)-CFRP, small pits were observed within the interface area near the perforation, which may have been a result of galvanic corrosion (Figure 12a). The pits were visible in the surrounding area where the carbon fiber was close to the surface, which may have created a local galvanic microcell. No such effect was observed in the laminate with glass fibers perpendicular to the carbon fiber (Figure 12b). The HSG interlayer, unobservable in such imaging, provided the metal–composite adhesion, with no delamination observable around the metal layer perforation areas. This indicated a considerably higher stability for this interlayer at limited chloride ion accessibility than during direct contact with an aggressive environment.

**Figure 13:**
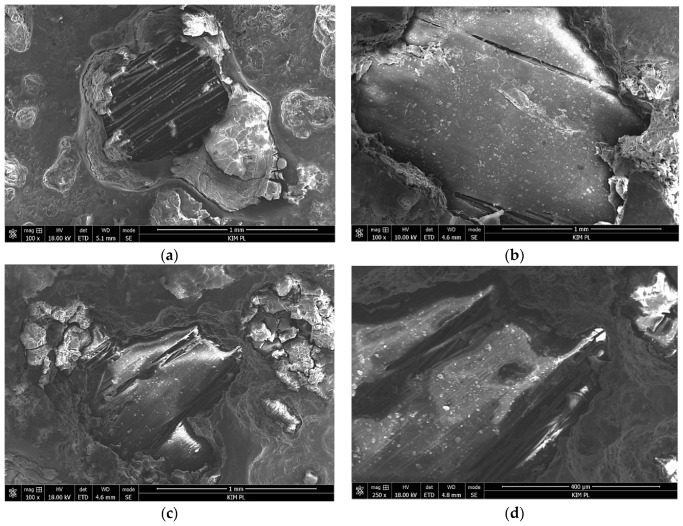
Microstructure in the pitting area: (**a**) Al-CFRP, (**b**) Al-GFRP(90)CFRP, (**c**) and (**d**) Al-GFRP(0)-CFRP; salt chamber 0.8 M NaCl for 12 weeks; SEM.

SEM observation of the pitting area in laminates after 12 weeks of soaking revealed various levels of composite degradation (Figure 13). In the Al-CFRP, degradation of the polymer matrix occurred in some pits, and carbon fibers were exposed (Figure 13a). In the Al-GFRP(90)-CFRP, the composite was not damaged in most pits, even in larger ones, and the smooth polymer surface was visible (Figure 13b). In some pits in the Al-GFRP(0)-CFRP, the polymer matrix of the glass composite was affected, with fibers exposed locally (Figure 13c,d). This observation suggested that the presence of a thin GFRP layer and its configuration could impact the course of laminate degradation once an aggressive environment reached the metal–composite interface.

## 4. Summary and Conclusions

The purpose of the presented tests was to determine the possibility of replacing the process of anodizing aluminum sheets for bonding with composites in FMLs with the sol–gel treatment in the context of corrosion resistance. The applied commercial formulation is used for adhesive bonding of aluminum with other materials and is recommended for use with a primer containing strontium chromate. In FMLs, aluminum sheet corrosion and metal–composite interface degradation may be initiated by humidity entering through poorly secured edges. The conductivity exhibited by carbon fibers may be an additional factor. Owing to the negligible corrosion rate of laminates fully covered with a protective coating, accelerated corrosion tests were selected, where direct contact took place between the HSG coating and an environment containing chloride ions. This allowed an assessment of the durability of the coating in an aqueous solution of NaCl and the observation of the Al(HSG)-GFRP and Al(HSG)-CFRP interface in areas surrounding metal perforation zones resulting from pitting corrosion.

The following observations were made:

1. An HSG coating does not provide sufficient protection against corrosion for an aluminum alloy in direct contact with an aggressive environment, but it is effective as an interlayer.
2. Impedance spectra indicate the complex structure of the coating that contains a reactive epoxy-silane layer and a conversion Al-O-Zr interlayer. After long-term contact with an environment containing chloride ions, water penetrates the porous structure of the cured sol–gel, and hydrolysis may occur. Migrating chloride ions react with the conversion interlayer and lead to the initiation of pitting corrosion in the metal.
3. Local aluminum layer perforation does not lead to delamination at the metal–composite interface despite the type or configuration of the composite. This confirms the durability of the HSG used in FMLs.

## Figures and Tables

**Table 1:** Precursors for the preparation of AC 130-2 sol–gel [25,27].

Abbreviation	Molecular Formula	Structural Formula
GPTMS	C_9_H_20_O_5_Si	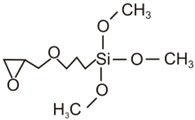
TPOZ	C_12_H_28_O_4_Zr	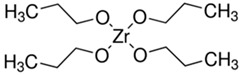

GPTMS, 3-glycidoxypropyl-trimethoxysilane; TPOZ, tetra-n-propoxyzirconium (zirconium(IV) propoxide).

**Table 2:** Thickness and electrochemical parameters of sol–gel coatings (mean values).

Sol–Gel Coating	Thickness Layer [μm]	*E*_OCP_ [V_SCE_]		*E*_corr_ [V_SCE_]		*i_c_*_orr_ [mAcm^−2^]		*R*_p_ [kΩcm^2^]	
		0.5 M NaCl	0.8 M NaCl	0.5 M NaCl	0.8 M NaCl	0.5 M NaCl	0.8 M NaCl	0.5 M NaCl	0.8 M NaCl
1 layer	0.5 ± 0.2	−0.52 ± 0.01	−0.57 ± 0.04	−0.52 ± 0.02	−0.57 ± 0.02	0.39 ± 0.09	0.69 ± 0.1	66.7 ± 8.5	37.7 ± 5.3
2 layers	0.8 ± 0.16	−0.53 ± 0.02	−0.56 ± 0.02	−0.52 ± 0.01	−0.54 ± 0.03	0.35 ± 0.08	0.39 ± 0.12	74.3 ± 7.2	66.7 ± 6.8
3 layers	1.2 ± 0.2	−0.55 ± 0.02	−0.56 ± 0.03	−0.49 ± 0.02	−0.54 ± 0.02	0.018 ± 0.01	0.044 ± 0.02	1444 ± 152	591 ± 75
